# Switching to a 'nuke-sparing' raltegravir/atazanavir combination: an individualised approach

**DOI:** 10.1186/1758-2652-13-S4-P33

**Published:** 2010-11-08

**Authors:** A Grant, Y Kamuntu, P Read, R Kulasegaram

**Affiliations:** 1 Guy's & St. Thomas' NHS Foundation Trust, Pharmacy, London, UK; 2 Guy's & St. Thomas' NHS Foundation Trust, Genitourinary Medicine & HIV, London, UK

## Introduction

The success of HIV treatment is limited by tolerability/toxicity of ARVs and patient adherence remains paramount to achieve viral suppression. Several small studies have investigated the combination Raltegravir and Atazanavir. This is particularly attractive to exclude NRTIs and ritonavir in metabolic toxicity. Optimum dosing has yet to be decided however recent data from SPARTAN suggests once daily regimens are associated with raltegravir resistance.

## Objectives

To assess HIV virologic control in patients switched to RAL/ATZ. To compare metabolic parameters before and after switch.

## Methods

We retrospectively identified patients RAL/ATZ from pharmacy database. Using medical notes and lab results system, we recorded the HIV-1 VL and lipid profiles pre- and post- switch. TDM, hepatitis status, drug resistance and treatment experience were also recorded.

## Results

We identified 11 patients on RAL/ATZ, 9 male. At switch, 1 patient had a detectable VL (582 copies/mL), all other patients were undetectable. At the most recent appointment all patients had an undetectable viral load. The mean number of previous regimens was 5; no patient commenced the regimen with known PI resistance. In 5 patients other ARVs were included, and in 6 RAL/ATZ were used alone.

Therapeutic drug levels of raltegravir and atazanavir were measured in 9/11 patients and the predicted trough levels for both raltegravir and atazanavir were greater than the minimum recommended concentration. There was a reduction in total cholesterol and triglycerides post switch and a trend towards reduction in LDL-cholesterol and total cholesterol:HDL ratio. Two patients were lost to follow up and three patients discontinued the combination. The reasons for discontinuation: to avoid drug interactions pre-renal transplant, to aid adherence (once daily regimen) and presumed raltegravir intolerance (anxiety and sleep disturbance).

## Conclusions

A switch was made when 10/11 patients were suppressed and required therapy change because of another reason. All patients maintained a viral load of less than 50 copies/ml (7-102 weeks).The combination was well tolerated and there was a trend towards improved lipid parameters observed. In the absence of resistance, this combination shows promise in terms of prolonged virologic efficacy, tolerability and an improved metabolic profile.

